# Multifocal micronodular pneumocyte hyperplasia with a novel mutation in *TSC1*: a case report

**DOI:** 10.3325/cmj.2021.62.523

**Published:** 2021-10

**Authors:** Ai Li, Rong Jiang, Yongxia Li, Xixian Teng, Kaijun Ding, Bingqian Yang

**Affiliations:** 1 Department of Respiratory and Critical Care Medicine, Second Affiliated Hospital of Kunming Medical University, Kunming, China; 2 Kunming Medical University, Kunming, China; 3 Second Affiliated Hospital of Kunming Medical University, Kunming, China; * The first two authors contributed equally.

## Abstract

We report on a 34-year-old woman diagnosed with tuberous sclerosis complex. The patient was admitted for respiratory manifestations, while multi-organ involvement made the diagnostic process challenging. Genetic testing revealed a novel mutation *TSC1* c.2094_2110del (p.His699Ter), which expands the disease-causing variant spectrum. Our results may facilitate the disease diagnostics and help to devise genetic counseling and targeted gene therapy.

## Case report

**Figure 1:**
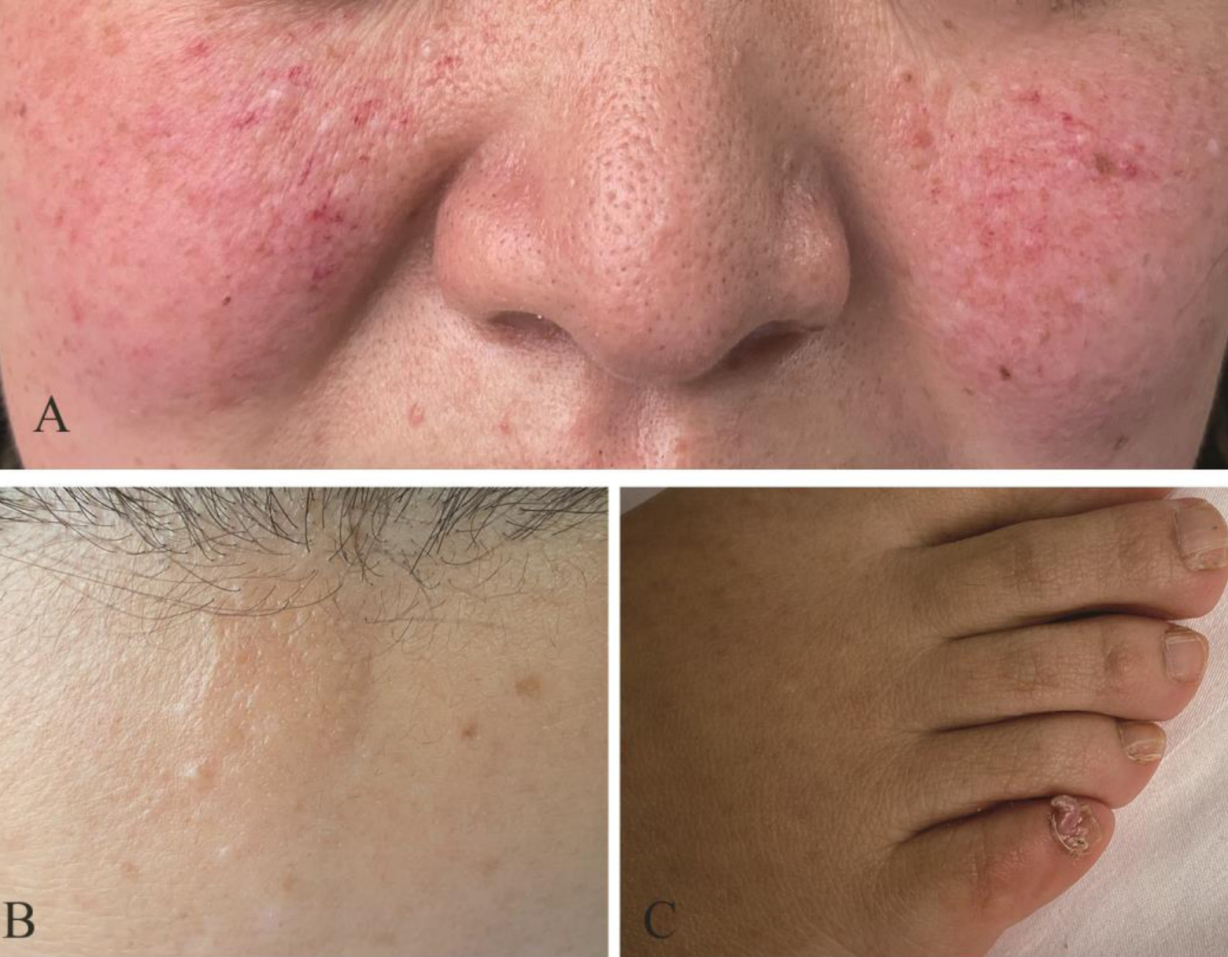
Skin lesions of the patient with tuberous sclerosis complex. (**A**) Multiple facial angiofibromas. (**B**) Fibrous cephalic plaque. (**C**) Subungual fibroma.

A 34-year-old woman presented with productive cough lasting for two months. She denied fever, dyspnea, chest pain, hemoptysis, weight loss, and extrapulmonary symptoms. No history of seizures or developmental delay was reported. The results of physical examinations were unremarkable, expect for skin evaluation, which revealed erythematous papules on the cheeks consistent with facial angiofibromas (Figure 1A), fibrous cephalic plaque (Figure 1B), and subungual fibroma (Figure 1C). Her family members did not exhibit any similar dermatologic manifestations.

**Figure 2:**
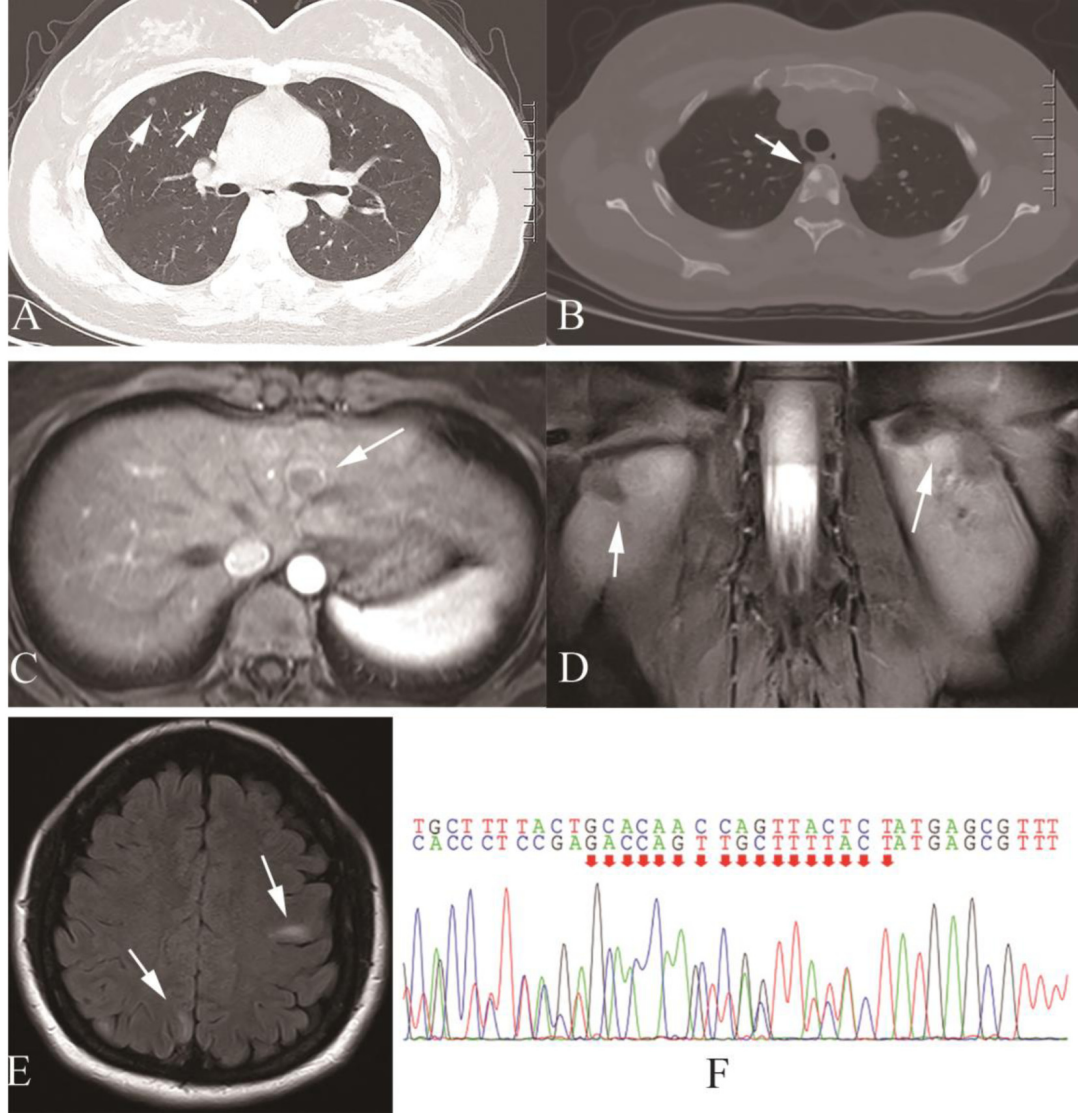
(**A**) Chest computed tomography (CT) revealed multifocal micronodular pneumocyte hyperplasia. (**B**) Thoracic spine CT revealed sclerotic bone lesions. (**C**, **D**) Abdomen magnetic resonance imaging (MRI) revealed angiomyolipoma on both kidneys and the left liver lobe. (**E**) Fluid attenuated inversion recovery brain MRI revealed cortical tubers. (**F**) Chromatogram confirmation indicated c.2094_2110del (p.His699Ter) mutation in *TSC1.*

Complete blood count and liver and kidney function tests were unremarkable. Computed tomography (CT) of the chest revealed multiple small and ground-glass nodules randomly distributed in both lungs, which supported the diagnosis of multifocal micronodular pneumocyte hyperplasia (MMPH) (Figure 2A). A CT scan of the thoracic spine revealed multiple sclerotic bone lesions (SBLs) (Figure 2B). Subsequent abdomen magnetic resonance imaging (MRI) showed multiple variable-sized masses on both kidneys and the left liver lobe consistent with angiomyolipoma (Figure 2C, 2D). MRI revealed multiple nodular lesions in the brain, strongly suggesting cortical tubers (Figure 2E). Pulmonary function test, electrocardiography, electroencephalography, and retinal examination all yielded unremarkable results.

TSC was diagnosed by clinical evaluation according to the latest criteria from 2019 (1). Genetic tests were performed on the patient’s request. Genomic DNA was isolated from peripheral blood leukocytes, and direct sequencing was performed. A novel heterozygous nonsense mutation c.2094_2110del (p.His699Ter) in *TSC1* was detected (Figure 2F).

**Figure 3:**
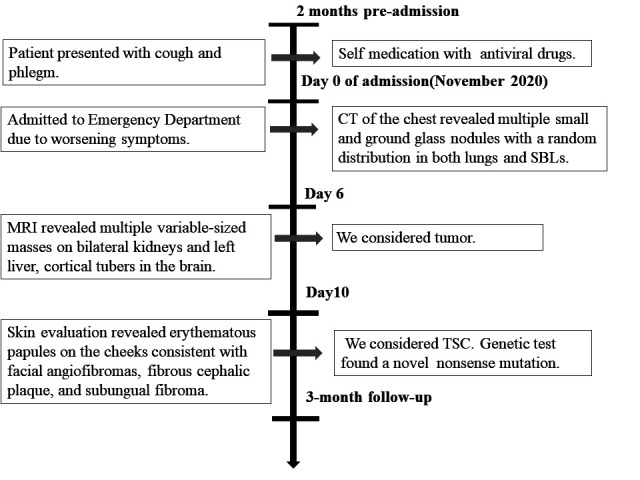
The timeline of the diagnostic process. CT – computerized tomography; SBLs – sclerotic bone lesions; MRI – magnetic resonance imaging; TSC – tuberous sclerosis complex.

The patient remained untreated because she did not have any symptoms and signs. At three-month follow-up, no disease progress was noted, and the patient did not suffer from productive cough. A timeline of the events is shown in Figure 3.

## Discussion

TSC is a rare autosomal dominant syndrome related to *TSC1* or *TSC2* mutations (2). It is a neurocutaneous disorder characterized by the presence of benign tumors in multiple organs, including the skin, brain, kidney, lung, and occasional malignant tumors (1,3). *TSC1* gene, encoding a protein named harmatin, is located on the chromosome 9q34 (4). Approximately 20% of TSC patients have *TSC1* mutations (5). TSC1 patients suffer from less severe manifestations than TSC2 patients (6). To the best of our knowledge, this is the first report of *TSC1* c.2094_2110del (p.His699Ter) variant associated with MMPH. This nonsense mutation may destroy the mammalian target of rapamycin pathway, reducing or even eliminating cell growth inhibition, proliferation, autophagy, and protein and lipid synthesis (7).

Clinical manifestations of TSC in the lungs include lymphangioleiomyomatosis, MMPH, and rarely a clear cell lung tumor (3). The estimated MMPH prevalence in patients with TSC is 40%-60% (8). MMPH is observed in both men and women with TSC (8). It is characterized by multiple solid nodules or nodular ground-glass opacities (GGOs) observed on CT, randomly distributed throughout the lungs (9). Histologically, MMPH refers to multicentric and well-demarcated nodular growth of type II pneumocytes (10). Therefore, the differential diagnosis should consider atypical adenomatous hyperplasia, adenocarcinoma, pulmonary metastases, tuberculosis, sarcoidosis, and histiocytosis X.

Given the imaging findings of diffuse pulmonary nodular GGOs, we considered malignancy, pulmonary metastases, and viral and tuberculosis infections. The patient denied exposure to infectious diseases. In order to confirm the diagnosis, we performed CT and MRI of other organs. Based on the imaging findings of the brain, bones, liver and kidney, our patient was initially misdiagnosed with a tumor. However, a physical examination performed before the biopsy revealed skin rashes indicative of TSC. This case-report presents valuable information regarding the diagnostic process of this difficult-to-diagnose disease. TSC diagnosis is especially challenging when the disease affects multiple organs and in patients admitted for respiratory manifestations. TSC disease should be considered in the presence of pulmonary nodular GGOs on CT. We also reported on a novel mutation in *TSC1*, which expands the disease-causing variant spectrum. Our results may facilitate the development of genetic counseling and targeted gene therapy.
